# Notch Is Required for Neural Progenitor Proliferation During Embryonic Eye Regrowth

**DOI:** 10.3390/ijms26062637

**Published:** 2025-03-14

**Authors:** Dylan J. Guerin, Belen Gutierrez, Baoyi Zhang, Kelly Ai-Sun Tseng

**Affiliations:** School of Life Sciences, University of Nevada, Las Vegas, NV 89154, USA

**Keywords:** retina, regeneration, Notch, *Xenopus*, regrowth, neural stem cells, RPCs, V-ATPase, eye

## Abstract

The ability of an organism to regrow tissues is regulated by various signaling pathways. One such pathway that has been studied widely both in the context of regeneration and development is the Notch signaling pathway. Notch is required for the development of the eye and regeneration of tissues in multiple organisms, but it is unknown if Notch plays a role in the regulation of *Xenopus laevis* embryonic eye regrowth. We found that Notch1 is required for eye regrowth and regulates retinal progenitor cell proliferation. Chemical and molecular inhibition of Notch1 significantly decreased eye regrowth by reducing retinal progenitor cell proliferation without affecting retinal differentiation. Temporal inhibition studies showed that Notch function is required during the first day of regrowth. Interestingly, Notch1 loss-of-function phenocopied the effects of the inhibition of the proton pump, vacuolar-type ATPase (V-ATPase), where retinal proliferation but not differentiation was blocked during eye regrowth. Overexpression of a form of activated Notch1, the Notch intracellular domain (NICD) rescued the loss of eye regrowth due to V-ATPase inhibition. These findings highlight the importance of the Notch signaling pathway in eye regeneration and its role in inducing retinal progenitor cell proliferation in response to injury.

## 1. Introduction

The ability of an organism to regrow lost or damaged tissues varies greatly among animals [1,2,3,4,5,6,7,8,9,10]. To understand why some animals, or even some tissues within otherwise regenerative animals, lack this ability, there is a need to understand the molecular mechanisms that regulate regrowth. An important model organism for studying regeneration is the African clawed frog, *Xenopus laevis*. *X laevis* has long been studied as a regenerative model, valuable for its external fertilization and development, large clutch sizes, and relatively rapid development time, the speed of which can be manipulated by ambient temperature regulation [11,12,13].

*Xenopus* displays age-dependent regeneration. Tadpoles can regrow a number of structures including the tail, limb, retina, and lens [3,14,15,16,17,18] with this ability generally decreasing in potency in some tissues as the animals age [19,20,21]. Our previous work showed that *Xenopus* tailbud embryos regrew their eyes following surgical ablation of ~85% of the tissues (including the lens placode and most of the optic cup) at the developmental stage (st.) 27 [10]. The eye completes regrowth within 5 days, is functional, and contains the normal complement of cell types [10,22]. The regrowth process requires an increase in cell proliferation and recapitulates retinogenesis. One advantage of this model is that eye regrowth in the embryo occurs concurrently with normal eye development on the uninjured contralateral side. In models where regrowth occurs post-development, comparison of eye regrowth and development can be challenging due to inherent differences between developing and mature tissues [17,23,24,25,26]. The embryonic eye regrowth model provides the opportunity for a more direct comparison between developmental and regrowth mechanisms regulating regrowth [19,27,28]. Therefore, it is important to understand whether and how developmental mechanisms act as regulators of regrowth.

An important regulator of eye development is the Notch signaling pathway. Notch is a transmembrane receptor that, upon binding to its ligand, undergoes cleavage events resulting in the cleavage of the intracellular domain (NICD), which migrates to the nucleus and acts as a transcription factor to regulate downstream target genes [29]. The Notch signaling pathway is a highly conserved and well-characterized developmental pathway that often determines if a cell population will proliferate or differentiate and, in some contexts, can maintain stem cell populations [30,31,32,33,34].

Notch signaling can also function as a regulator of stem cell proliferation. In the *Drosophila* wing disc, Notch works to regulate proliferation, and overexpression of the Delta ligand is sufficient to increase proliferation in the wing disc [35]. In mice, Notch promotes proliferation and maintains stemness in intestinal crypt base columnar stem cells. A reduction in Notch signaling reduces the proliferation and expression of stem cell–specific markers and promotes differentiation [36]. Similar behavior is found in bone marrow mesenchymal stem cells, where inhibition of Notch1 signaling results in reduced proliferation [37].

During *Xenopus* tadpole tail regeneration, Notch signaling is required for proper regrowth. Following tail amputation, treatment with MG132 (a proteasome inhibitor that blocks the cleaving of the Notch1 protein) results in the healing of the tail stump without regeneration [19]. Additionally, during the refractory period—when the tadpole temporarily loses its tail regenerative ability—activation of Notch signaling stimulates tail regeneration following amputation [19,38]. During *Xenopus* eye development, active Notch serves to maintain a pool of multipotent retinal progenitor cells (RPCs) by regulating cell differentiation [39]. An imbalance in Notch activity during development results in eye malformations [39,40]. Given the known roles of Notch in regulating stem cell populations and promoting appendage regeneration, it is likely that Notch acts to regulate RPC proliferation during *Xenopus* eye regrowth.

Here, we investigate the role of Notch signaling in *Xenopus* embryonic eye regrowth. Our study shows that the loss of the Notch1 function blocks eye regrowth and results in small eyes. Notch inhibition reduces RPC proliferation but does not block retinal differentiation. We also determine that the activation of Notch1 during eye regrowth rescues regrowth-inhibited small-eye phenotype caused by V-ATPase inhibition, demonstrating a link between Notch and V-ATPase signaling. Together, our results show that Notch1 is required for eye regrowth.

## 2. Results

### 2.1. Reduction in Notch Signaling Following Eye Ablation Inhibits Regrowth

Successful eye regrowth requires the complex interaction of multiple cell signaling pathways, including bioelectrical signaling and apoptosis [10,21,41]. Another candidate pathway is the Notch signaling pathway, which is required for the development of the eye [42,43,44]. In *Xenopus*, Notch1 can act as a neural stem cell marker. During development, it is expressed widely in the optic cup and inhibits retinal differentiation [30,45]. However, the role of Notch1 in neural regrowth is unclear. Thus, we investigated whether Notch1 is required for eye regrowth.

**Figure 1:**
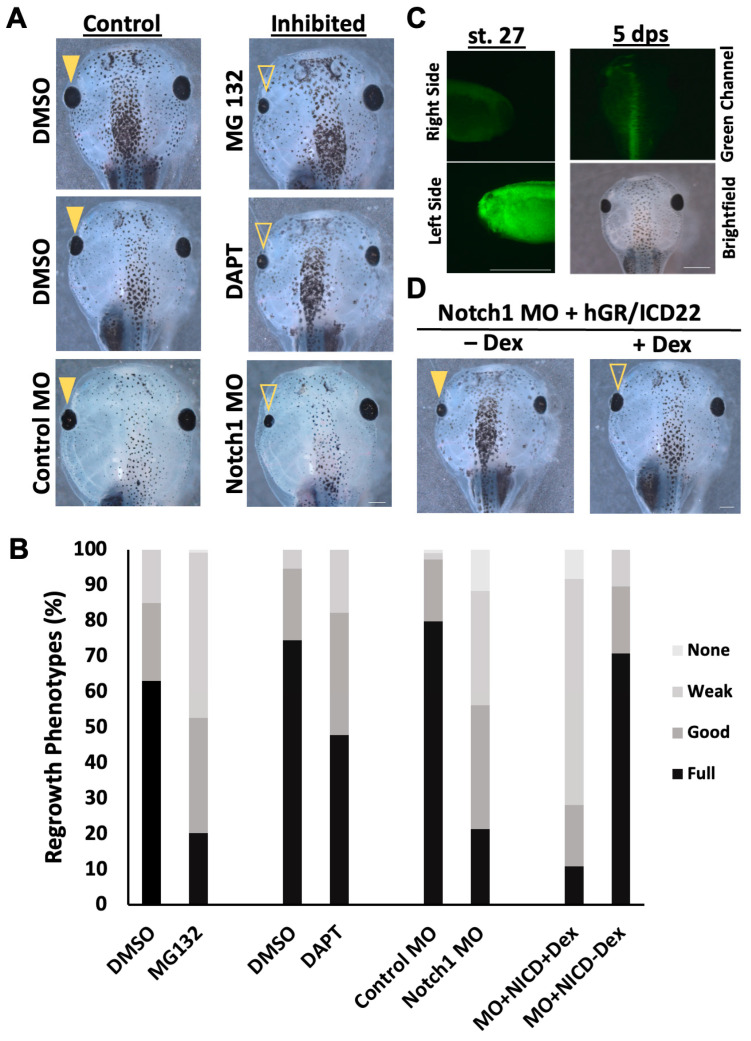
Reduced Notch impairs eye regrowth. (**A**) Comparison of 5 dps tadpoles treated with DMSO (control), Notch inhibitors (MG132 or DAPT), or injected with Notch morpholino (n > 30 per condition). Images at 20.5×. Up = anterior. (**B**) Graph showing the percentage of the population achieving full regrowth at 5 dps. (**C**) Morpholino expression at st. 27 and 5 dps. Cells containing morpholino showed green fluorescence due to the fluorescein-tagged oligonucleotide. The top left panel shows the right side of the embryo, right = anterior. The bottom left panel shows the left side of the same embryo, right = posterior. For both, up = dorsal. Images at 48×. The tadpole at 5 dps still exhibits green fluorescence on the left side of the animal. The top right panel is a green channel showing the fluorescein tag on the morpholino at 5 dps, showing the persistence of morpholino throughout the regrowth period. The bottom right panel is the corresponding brightfield image. Up = anterior. Image at 25×. Scale bar = 500 μm. (**D**) Comparison of 5 dps tadpoles expressing the Notch1 morpholino and the Dex-inducible NICD construct in the left eye region. The left panel shows regrowth without the Dex inducer; the right panel shows regrowth with Dex. The closed arrowhead indicates a control regrowth-inhibited eye. The open arrowhead indicates rescued eye growth (n > 30 per condition). Images at 20.5×. Up = anterior, down = posterior. (**A**,**C**,**D**) Scale bar = 500 μm.

First, we sought to inhibit Notch signaling during eye regrowth. Numerous studies on Notch function in various tissues have successfully utilized the cysteine protease inhibitor MG132, as well as the γ-secretase inhibitor DAPT, as Notch inhibitors by blocking the cleavage of the intracellular domain of the Notch protein, thereby restricting downstream activation [3,38,46,47,48,49,50,51,52]. We first titrated each inhibitor to identify dosages that enabled normal development and used these concentrations (10 µM MG132 and 5 µM DAPT) for our experiments. Then, we carried out eye regrowth assays to observe the effects of inhibitor exposure. Treatment with either chemical inhibitor following st. 27 ablation surgery caused a noticeable decrease in eye size compared to age-matched vehicle-treated regrowing controls (Figure 1A,B). We used the Regrowth Index (RI, ranging from 0 to 300; described in Methods) to assess the overall quality of regrowth as evaluated using eye size and morphology. A 10 µM MG132 treatment resulted in 20.2% of fully regrown eyes (RI = 172, n = 114) compared to DMSO (vehicle)-treated regrowing control, which resulted in 63% of fully regrown eyes (RI = 248, n = 100, *p* < 0.01). A 5 µM DAPT treatment resulted in 47.9% of fully regrown eyes (RI = 218, n = 96) compared to the DMSO-treated regrowing control, which resulted in 74.47% of fully regrown eyes (RI = 269, n = 94, *p* < 0.01) (Figure 1A,B). As chemical inhibitors can potentially have off-target effects, these results were confirmed using molecular inhibition.

A verified morpholino against *Xenopus* Notch1 mRNA [53,54,55] or a control morpholino was injected into the left dorsal blastomere of four-cell embryos at a concentration that did not affect embryogenesis. The morpholinos were tagged with fluorescein, allowing for the selection of embryos with eye regions that contained high levels of either the control or Notch1 morpholino (Figure 1C). At st. 27, eye ablation surgeries were performed on these embryos, and regrowth was assayed at 5 dps. Consistent with the chemical inhibition results using two different Notch inhibitors, embryos injected with the Notch1 morpholino showed significantly reduced eye regrowth (21.4% full regrowth, RI = 165, n = 109) compared to those injected with the control morpholino (79.8% full regrowth, RI = 276, n = 112, *p* < 0.01) (Figure 1A,B). To confirm that the morpholino effect was due to Notch1 knockdown, we tested if ectopic expression of Notch1 could restore eye regrowth. The Notch Intracellular Domain (NICD) is an activated form of Notch and an established tool to activate Notch signaling in *Xenopus* [30]. Dexamethasone-inducible NICD mRNA was co-injected with the Notch1 morpholino at the four-cell stage. After eye ablation surgery, 10 µM dexamethasone (Dex) was added to induce NICD expression. Dexamethasone treatment resulted in 59.1% full eye regrowth (RI = 248, n = 22) compared to 9.5% full regrowth in the uninduced control (RI = 144, n = 21; *p* < 0.01) (Figure 1B). Thus, NICD activation was sufficient to rescue Notch1 morpholino-inhibited regrowth (Figure 1D). This result indicates that it was the reduction in Notch1 that caused the inhibition of regrowth. Together, our data demonstrated that Notch is required for a successful regrowth of the eye.

### 2.2. Notch Is Required During the First Day of Regrowth

Notch signaling is often a regulator of proliferation [35,36,56]. Our previous work showed that approximately 87% of the increase in eye size during regrowth occurred during the first two days [10]. We hypothesized that the requirement for Notch function during eye regrowth is confined to the early time period. In the eye regrowth model, treatment with MG132 inhibition gave stronger regrowth inhibition compared to DAPT (Figure 1).

**Figure 2:**
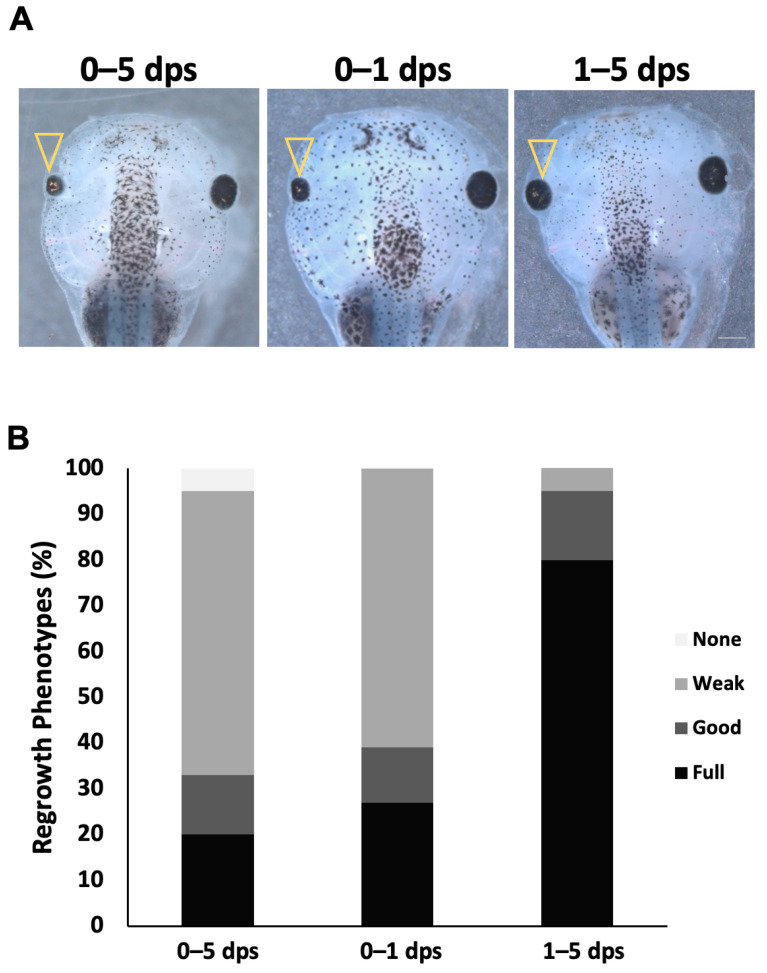
Notch is required during the first day of regrowth. (**A**) Comparison of regrown eyes at 5-dps following treatment of MG132 for variable durations from the time of surgery (n > 30 per condition). Open arrowheads indicate a regrown eye. Up = anterior, images at 20.5×. Scale bar = 500 μm. (**B**) Graphical representation of the percentage of regrowth phenotypes at 5 dps.

To identify the temporal requirement for Notch, the duration of exposure to 10 µM MG132 was varied during the regrowth period. Our data indicated that embryos with reduced Notch function during the first 24 h post-surgery resulted in similar inhibition (RI = 191, n = 99) with 25.3% of fully regrown eyes compared to embryos inhibited for the entire five-day period with 24.7% of fully regrown eyes (RI = 191, n = 93, *p* > 0.05) (Figure 2). If the key requirement for Notch activity is during the first day of regrowth, then its inhibition after 1 dps should not affect regrowth. Consistent with this prediction, embryos treated with MG132 from 1 dps through the end of the five-day assay showed no appreciable inhibition of regrowth, with 78.8% of fully regrown eyes, a level that was comparable to DMSO-treated controls (RI = 250, n = 92; *p* < 0.01 compared to either 0–5 dps or 0–1 dps treatment) (Figure 2B). Together, our data show that the first 24 h is the critical period during which Notch function is required to drive eye regrowth.

### 2.3. Retinal Differentiation Occurs During Notch Inhibition

Retinogenesis in *X. laevis* begins at st. 24 and is completed within 2 days (approximately st. 41). Eye regrowth occurs via an increase in cellular proliferation during the first day, while retinal differentiation is concomitantly delayed by one day [22]. Although retinogenesis occurs later than normal, the overall two-day retinogenesis period is maintained, similar to that of developing eyes. In order for eye regrowth to occur fully, both cell proliferation and proper retinal differentiation are needed. Previously, we showed that the regrown eye contained the expected complement of retinal cell types, including rod and cone photoreceptors, ganglion cells, retinal pigmented epithelium (RPE) cells, and Müller glia, with the same structure as a normally developing eye [10]. Defective regrowth could result from disruptions in retinal cell proliferation, differentiation, or both.

To characterize the regrowth defects resulting from Notch inhibition, we first examined the effects on retinal differentiation. It is possible that reducing Notch function during regrowth disrupted retinal differentiation, leading to small eyes. In order to test this, we examined retinal differentiation using known antibody markers. Embryos were fixed at 3 dps following either treatment with MG132 or injection with Notch1 morpholino, as well as in control regrowing eyes. Eye sections were obtained and stained with anti-Islet1 antibody as a marker of ganglion cells, anti-Rhodopsin antibody as a marker of rod photoreceptor cells, anti-Glutamine Synthetase antibody as a marker of Müller Glia cells, anti-Calbindin antibody as a marker of cone photoreceptor cells, or anti-RPE65 antibody as a marker of the retinal pigment epithelium [22].

**Figure 3:**
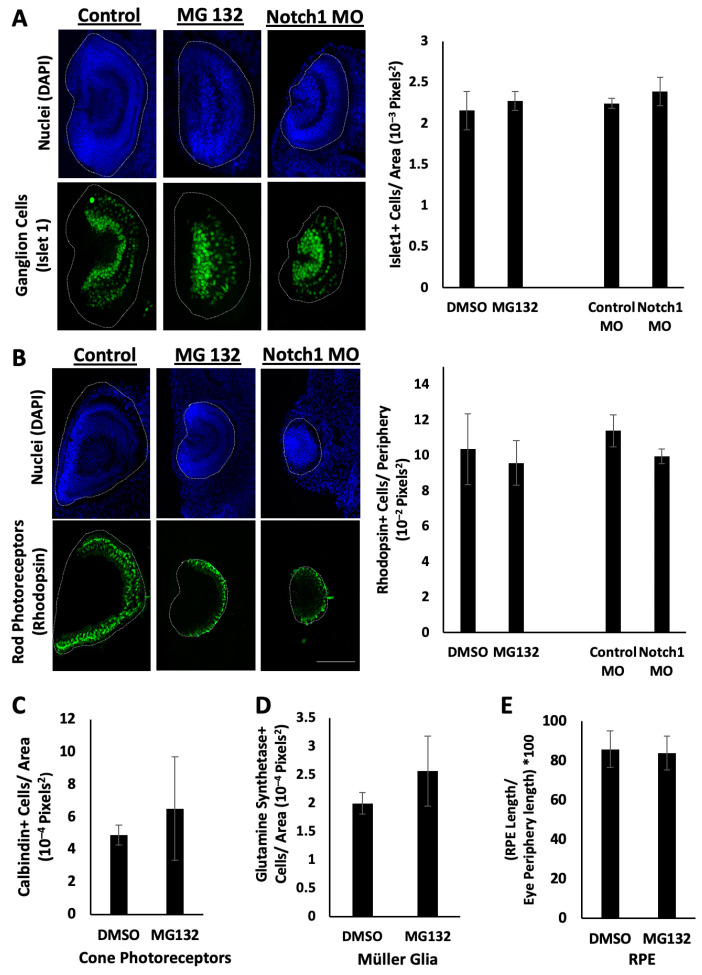
Retinal differentiation during Notch inhibition. (**A**) Transverse eye sections of regrowing and inhibited eyes at 3 dps. The top panels indicate nuclei. The bottom panels indicate ganglion and inner nuclear layer cells. The graph shows Islet1-positive cells per eye area. (**B**) Transverse eye sections of regrowing and inhibited eyes at 3 dps. The top panels indicate nuclei. The bottom panels indicate rod photoreceptor cells. The graph shows the number of Rhodopsin-positive cells along the periphery of the eye. Dotted lines outline the eye region. All eyes were sectioned medially facing left. (**C**) Graph comparing Calbindin-positive cells in control regrowing and Notch-inhibited eye. (**D**) Graph comparing Glutamine Synthetase-positive cells in control regrowing and Notch-inhibited eyes. (**E**) Graph comparing the percentage of the regrown eye periphery occupied by the RPE in control regrowing and Notch-inhibited eyes (n > 3 per condition, *p* > 0.05). Images at 20×. Scale bar = 100 μm. Up = dorsal and left = anterior.

Similar retinal patterns were observed between the Notch-inhibited, non-regrowing small eyes and the control regrowing eyes in terms of overall morphology and the positioning of retinal cell types, including ganglion cells and rod photoreceptor cells, relative to their eye sizes, with no statistical differences (Figure 3A,B). Islet1 signal was present within the ganglion cell layer and inner nuclear layer in both the control regrowing and Notch-inhibited non-regrowing eyes (Figure 3A, n > 5 for each condition), although the overall pattern appeared less organized compared to the control. As expected, the Rhodopsin signal spanned the posterior periphery of the eye, in the outer nuclear layer (Figure 3B, n > 5 for each condition). Quantification of the number of ganglion cells showed that they were represented in similar proportions after area normalization in the Notch-inhibited and normal regrowing eyes (n = 7, average of 2.16 × 10^−3^ pixel^2^ for DMSO control; n = 5; average of 2.27 × 10^−3^ pixel^2^ for MG132 Notch-inhibited, *p* = 0.10; n = 6; average of 2.24 × 10^−3^ pixel^2^ for control morpholino; n = 5; and average of 2.39 × 10^−3^ pixel^2^ for Notch1 morpholino, *p* = 0.17) (Figure 3A). Quantification of the number of rod photoreceptors showed that they were also represented in similar proportions in the Notch-inhibited and normal regrowing eyes (n = 8, average of 10.36 cells × 10^−2^ pixel^2^ along the periphery of the eye for DMSO control; n = 5, average of 9.58 cells × 10^−2^ pixel^2^ along the periphery of the eye for MG132 Notch-inhibited, *p* = 0.08; n = 7, average of 11.39 cells × 10^−2^ pixel^2^ along the periphery of the eye for control morpholino; and n = 6, average of 9.95 cells × 10^−2^ pixel^2^ along the periphery of the eye for Notch1 morpholino, *p* = 0.18) (Figure 3B).

Comparable results were observed for other retinal cell types: cone photoreceptor cells (n = 4, average of 4.89 cells × 10^−4^ pixel^2^ for DMSO control, and n = 5, average of 6.52 cells × 10^−4^ pixel^2^ for MG132 Notch-inhibited, *p* = 0.38) (Figure 3C); and Müller Glia (n = 3, average of 2.00 cells × 10^−4^ pixel^2^ for DMSO control, and n = 3, average of 2.56 cells × 10^−4^ pixel^2^ for MG132 Notch-inhibited, *p* = 0.34) (Figure 3D). Quantification of the RPE in regrown and Notch-inhibited eyes showed similar proportions of RPE length relative to the overall circumference of the eye (n = 3, average RPE length 85.8% of the periphery of the eye for DMSO control, and n = 7, average of 84.0% of the periphery of the eye for MG132 Notch-inhibited, p = 0.80) (Figure 3E). These observations are consistent with previous studies in Xenopus showing that abnormal small eyes can contain the general organization of the retinal layers, although disorganization may be observed [22,57]. Additionally, our data suggested that regrowth-inhibited eyes largely complete retinal differentiation by 3 dps (Figure 3), the same as seen for regrowing eyes. Together, our findings indicate that retinal differentiation proceeds when the Notch function is reduced.

### 2.4. Inhibition of Notch Function Downregulates Retinal Proliferation

In order for the eye to regrow successfully, cellular proliferation must take place to replace the tissues that were lost. There is a significant increase in the number of proliferating cells in the regrowing eye during 0–1 dps relative to normally developing eyes [10]. This increase is within the same window in which Notch is required for eye regrowth. Notch is known to play a role in regulating proliferation during eye development [58]. As Notch does not appear to regulate retinal differentiation during regrowth, we examined whether Notch regulates cell proliferation during regrowth.

**Figure 4:**
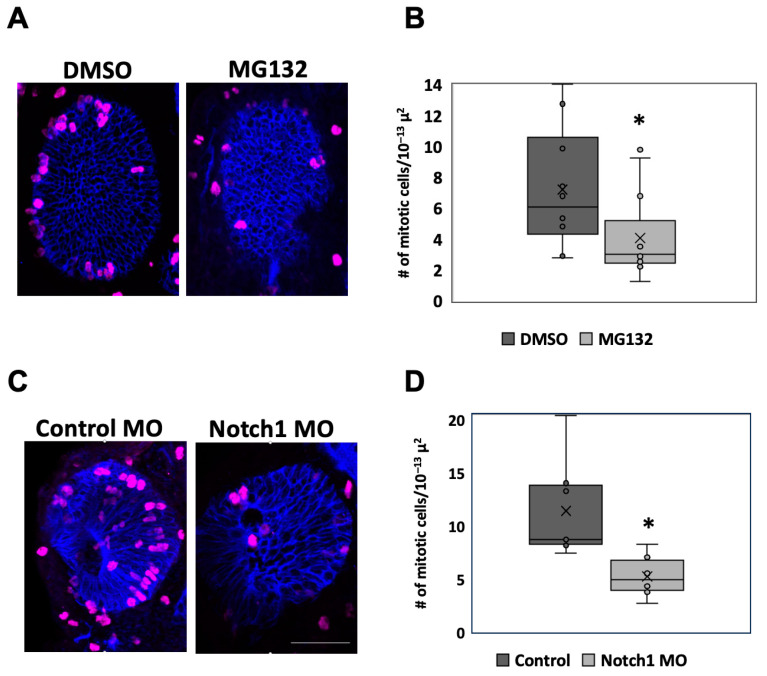
Reduction in Notch during eye regrowth reduces proliferation. (**A**) Transverse eye sections at 1 dps showing mitosis cells in DMSO or MG132-treated eyes (n > 5 per condition). Magenta = phosphorylated Histone 3, Blue = Xen1. Images at 20×. (**B**). Box and whisker plot comparing the number of H3P-positive cells normalized to area. (**C**) Transverse eye sections at 1 dps comparing proliferating cells in control morpholino and Notch1 morpholino injected eyes (n > 5 per condition). Magenta = phosphorylated Histone 3, Blue = Xen1. Images at 20×. Scale bar= 100 μm. (**D**) Box and whisker plot comparing number of H3P-positive cells normalized to area. * = *p* < 0.01. Up = dorsal and left = anterior.

Embryos injected in the left dorsal blastomere at the four-cell stage with Notch1 morpholino or treated with MG132 following surgery were fixed at 1 dps, sectioned, and stained with anti-phospho-Histone H3 (H3P) antibody, an established marker of mitosis [41,59,60]. The number of H3P-positive cells was counted and then normalized to the area of the eye to account for the decreased size of the regrowth-inhibited eyes (Figure 4A,C). Consistent with morphological observations (Figure 3A,B), embryos with chemical (n = 13) or molecular (n = 9) Notch inhibition showed either 56.7% or 45.8% reduction in the number of mitoses relative to their respective control regrowing eyes (n = 10 and n = 8; *p* < 0.05) (Figure 4B,D). These data indicate that a reduction in Notch during the regrowth period led to a reduction in RPC proliferation within the eye during regrowth.

### 2.5. Notch1 Overexpression Restores Eye Regrowth During V-ATPase Inhibition

Although much attention has focused on the roles of well-characterized developmental signaling pathways in regrowth, there are other key mechanisms that also determine regenerative success. Our work showed that the proton pump V-ATPase is required for eye regrowth but does not appear to have a role in eye development [41]. In mice, blockage of V-ATPase activity reduced Notch signaling, leading to reduced proliferation of neural stem cells [61]. In *Xenopus*, V-ATPase and Notch1 functions are required individually during the first day of regrowth ([41] and Figure 2), during a period of increased cell proliferation and deficient differentiation. We further observed that the block in eye regrowth caused by Notch inhibition resulted in similar phenotypes as the effects of V-ATPase inhibition: small eyes and reduced cell proliferation, with consistent retinal patterning. As such, we asked whether Notch and V-ATPase can interact to regulate eye regrowth.

**Figure 5:**
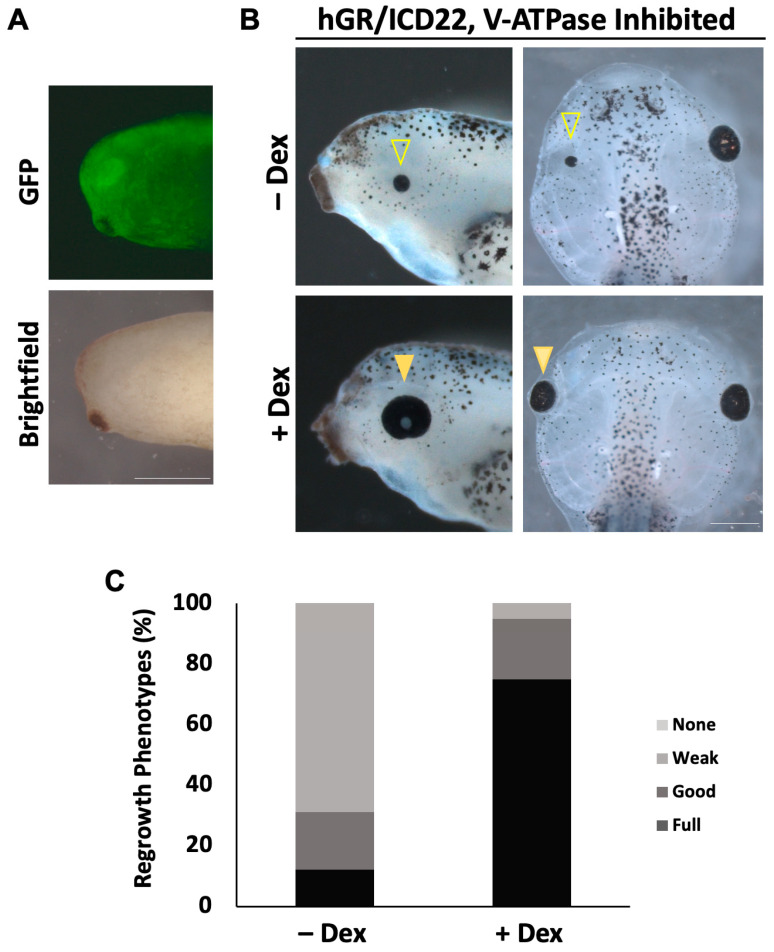
Notch1 overexpression during regrowth rescues V-ATPase inhibition. (**A**) Notch1 RNA is present in the eye at the time of surgery. Image of a st. 27 embryo head injected at st. 3 with GR-NICD RNA and GFP RNA. Right = posterior, left = anterior, up = dorsal, and down = ventral. Images at 48×. (**B**) Comparison of tadpoles at 3 dps (**left**) and 5 dps (**right**) treated with Concanamycin, injected with Dex-inducible NICD at st. 3, and treated with or without inducer immediately following ablation. Closed arrowhead indicates control regrowth-inhibited eye. Open arrowhead indicates rescued regrown eye through NICD overexpression. n > 30 per condition. Images at 25×. Scale bar = 500 μm. (**C**) Graph showing the percentage of the population achieving full regrowth at 5 dps with or without NICD activation. Up = dorsal and left = anterior.

To test whether V-ATPase inhibition of regrowth can be restored through ectopic activation of Notch signaling, we co-injected the following: GFP mRNA along with mRNA for Dex-inducible NICD into the left dorsal blastomere at the four-cell stage; and later selected for those embryos expressing GFP in the left eye region (Figure 5A). To inhibit V-ATPase activity, the highly specific inhibitor Concanamycin A was used [41,62,63]. A concentration of 20 nM Concanamycin A successfully blocked eye regrowth without affecting development [41]. Following eye ablation, embryos were treated with Concanamycin A and 10 µM dexamethasone, allowing for the ectopic activation of Notch in the eye region after surgery. Controls were treated with Concanamycin A only. Regrowth was assessed at 5 dps (Figure 5B,C). Control non-induced embryos treated with Concanamycin A resulted in 22.6% of fully regrown eyes with a low RI of 131 (n = 110). In contrast, embryos expressing NICD in the presence of Concanamycin A resulted in 75.5% of fully regrown eyes with an RI of 260 (n = 106, *p* < 0.01), representing a comparable quality of regrowth to untreated regrown eyes. Our data show a significant rescue of V-ATPase inhibition phenotype in embryos overexpressing NICD compared to the control. This result shows that activation of Notch restored eye regrowth following V-ATPase inhibition.

## 3. Discussion

In this study, we show that Notch1 is a required component for successful eye regrowth in *Xenopus laevis*. This finding is consistent with previous studies linking well-known eye developmental pathways, such as FGF, Pax6, retinoic acid, Wnt, and JAK/STAT, as necessary for successful retinal regeneration [8,22,64,65,66,67,68,69,70,71]. During *Xenopus* eye formation, Notch promotes RPC proliferation by inhibiting differentiation [39]. Notch is also active in the ciliary margin zone (a self-renewing proliferative region located at the periphery) of the mature tadpole retina and acts to maintain retinal progenitor cells [72]. We determined that Notch signaling increased retinal progenitor proliferation during regrowth. Although Notch can display pleiotropic effects, its function did not appear to play a major role in retinal differentiation during regrowth. This is consistent with the observation that Notch is required only during the first day of eye regrowth but not later on (Figure 2) when delayed retinogenesis becomes active.

In *Xenopus*, retinal differentiation starts at st. 24 and is completed by st. 41, over a period of two days. At st. 27, RPC cell division time is 8.6 h, and it increases to 56 h by st. 37/38, when most cells have exited the cell cycle [73]. In other types of neural stem cells, inhibition of Notch signaling has been shown to lengthen the cell cycle time [74,75]. These results suggest that reducing Notch during regrowth may lead to a lengthening of RPC doubling time with the consequence of a smaller RPC pool causing a failure to restore the eye to the appropriate size. In this case, the likely role of Notch in regrowth would be to maintain the short RPC doubling time (as in st. 27) to promote the restoration of the retinal progenitor population after eye ablation.

Multiple studies suggest a dynamic role for Notch signaling in retinal regeneration. After zebrafish retinal injury, the Müller glia responds by asymmetrically dividing to provide a neural progenitor cell population capable of regeneration [76,77,78,79]. Notch signaling is upregulated during regeneration but normally acts to maintain quiescence in adult Müller glia populations by downregulating proliferation when there is no damage [80]. Chick and rodent retinas undergo limited retinal regeneration. Inhibition of Notch signaling reduced progenitor cell proliferation [81,82,83]. However, continued Notch signaling subsequently prevented neuronal differentiation in the chick retina. Nevertheless, Notch signaling is consistently supportive of increasing progenitor pools during retinal regeneration, as observed in this study for *Xenopus* eye regrowth.

Like mammals, there are four Notch genes (Notch1–4) identified in *Xenopus*. In this study, we used a morpholino specific to *Notch1* [53]. However, DAPT and MG132 are expected to block all Notch family members; thus, a role for the other Notch homologues in eye regrowth cannot be ruled out. Aside from Notch1, very little is known about the other *Xenopus* Notch genes. Notch2 is expressed in the lens anlage at st. 28 [84], but its function has not been characterized. The roles of *Xenopus* Notch3 and Notch4 are unknown. Interestingly, a homologue of *Xenopus* Notch3 was found to be upregulated during goldfish retinal regeneration [85]. Investigating the roles of Notch2–4 may reveal additional functions for Notch signaling during eye development and/or regrowth.

The mechanisms that regulate eye regrowth are beginning to be identified [10,22,41]. Here, we found that ectopic expression of NICD rescued eye regrowth failure resulting from inhibition of V-ATPase. Other studies have observed similar interactions between V-ATPase and Notch. V-ATPase is expressed on cellular membranes as an essential H^+^ pump. In *Drosophila*, reduction in V-ATPase activity caused disruptions in endocytic acidity, leading to defective trafficking and processing of the Notch protein [86] V-ATPase inhibition in mice led to a reduction in Notch signaling, which could be rescued by the expression of NICD but not by a plasma membrane-bound form of activated Notch, suggesting that V-ATPase acts upstream of γ-secretase-dependent cleavage of the NICD [60]. The regulation of Notch processing by V-ATPase could be the mechanism that is used during eye regrowth. However, other findings suggest alternative mechanisms. The ectopic expression of a yeast plasma membrane H^+^ pump, PMA, was sufficient to rescue tadpole tail and eye regrowth failures induced by V-ATPase inhibition [41,58]. As PMA is located on the cell surface, this finding suggests that it is the plasma membrane functions of V-ATPase that are essential for eye regrowth rather than its vesicular membrane roles. During tadpole tail regeneration, Notch1 RNA expression in the regeneration bud is absent when bioelectrical signaling is inhibited [87]. Additionally, transcriptional profiling of *Drosophila* neuroblasts suggested that Notch and V-ATPase also interact in a regulatory loop [88]. Further studies will be needed to explore the specific interactions between Notch and V-ATPase in promoting eye regrowth.

Many developmental signaling pathways play an essential role in regrowth of tissues after injury. However, the specific interactions may not be the same during tissue restoration. As observed in development, we found that Notch promotes neural proliferation during eye regrowth [39,89,90]. We also showed that activation of Notch can bypass the requirement for V-ATPase activity in eye regrowth. Although Notch plays a key role in *Xenopus* eye development and is active at st. 27 [91], there is no known role for V-ATPase in the same process. Here, activation of V-ATPase appears to be a regrowth-specific signal. Therefore, it would be informative to individually determine which of the common developmental pathways are active during eye regrowth and how they interact with regeneration-specific mechanisms. Furthermore, this knowledge may help inform potential strategies for ocular repair.

## 4. Materials and Methods

Embryo culture and surgery: This study was carried out in accordance with the recommendations of the University of Nevada, Las Vegas Institutional Animal Care and Use Committee (IACUC). Embryos were obtained via in vitro fertilization and raised in 0.1X Marc’s Modified Ringer (1 mM MgSO_4_, 2.0 mM KCl, 2 mM CaCl_2_, 0.1 M NaCl, 5 mM HEPES, pH 7.8) [12]. Eye ablation surgery was performed as described in Kha et al., 2020 [92]. Following surgery, embryos were cultured at 22 °C.

Assessment of Eye Regrowth: The regrowth quality of eyes treated with chemical or molecular inhibitors after surgery was compared to age-matched regrown eyes from the same batch of embryos by calculating the Regrowth Index (RI), a quantitative measurement where the percentage of embryos achieving each category of regrowth is assigned a numerical value, with values for the group ranging from 0–300, with 0 indicating that all embryos failed to regrow eyes and 300 being all embryos fully regrew eyes, as described in [10]. The RI is based on four phenotype categories: full, partial, weak, and none. A fully regrown eye includes the same overall morphology and size as the unoperated, age-matched sibling. A partially regrown eye is visibly slightly smaller and may contain minor abnormalities. A weakly regrown eye is significantly smaller, malformed, and lacking the lens. When no regrown eye tissue is present, it is placed in the “none” category [92]. Regrowth experiments were performed on embryos from three different mothers with an n ≥ 30 for each mother per condition, for a total of n ≥ 90 embryos per condition. Embryos were pooled when calculating RI for each condition.

Chemical Treatments, and Morpholino and RNA Injections: Inhibitors were dissolved in DMSO. For knockdown of Notch, embryos were cultured in a medium containing DAPT (Cayman Chemical, Ann Arbor, MI, USA, CAS number 208255-80-5) or MG132 (Cayman Chemical, Ann Arbor, MI, USA, CAS number 1211877-36-9) immediately following surgery and cultured in the medium for five days. Control embryos were immersed in a medium containing an equivalent concentration of DMSO. To determine the temporal requirement for Notch, embryos were cultured in MG132 for varying time periods. As needed, embryos were removed from chemical-containing media, washed in 0.1× MMR, and transferred to 0.1× MMR or taken from 0.1× MMR at the 1 dps time point and transferred into media containing MG132 for the remainder of the regrowth period (4 days). At 5 dps, embryos were washed with fresh media, anesthetized, and regrowth was assayed.

For morpholino injections, the following published morpholinos were purchased from Gene Tools LLC (Philomath, OR, USA): Notch1 5′-GCACAGCCAGCCCTATCCGATCCAT-3′ [53] and the non-specific standard control oligomer: 5′-CCTCTTACCTCAGTTACAATTTATA-3′. Each morpholino contained a 3′ fluorescein addition. A total of 2.5 ng of morpholinos were injected into the left dorsal blastomere at the four-cell stage. Embryos with fluorescent signals in the eye region at st. 27 were selected for further analysis. hGR/ICD22 and GFP were transcribed *in vitro* from linearized plasmid constructs using the mMESSAGE Transcription Kit (Thermofisher, Waltham, MA, USA). For injections, GFP and 0.25 ng hGR/ICD22 [30] mRNAs were co-injected into the left dorsal blastomere at the four-cell stage. Embryos with fluorescent signals in the eye region at st. 27 were selected for further analysis. Dexamethasone was added to the media as an inducer at a final concentration of 10 μM.

Embryo Sectioning and Immunofluorescence Microscopy: For agarose embedding and sectioning, animals were fixed overnight at 4 °C in MEMFA (100 mM MOPS (pH 7.4), 2 mM EGTA, 1 mM MgSO_4_, and 3.7% (*v*/*v*) formaldehyde) [12] and dehydrated in methanol. After rehydration, embryos and tadpoles were embedded in 4–6% low-melt agarose and sectioned into 60 µm slices using a Leica vt1000s vibratome (Wetzlar, Germany). Sections were stained with primary antibodies, including Xen1 (pan-neural antibody, clone 3B1, 1:50 dilution, Developmental Studies Hybridoma Bank, RRID: AB_531871), anti-Islet1 (retinal ganglion cells and inner nuclear cell layer, clone 40.2D6, 1:200 dilution, Developmental Studies Hybridoma Bank, RRID: AB_528315), anti-Rhodopsin (rod photoreceptor cells, clone 4D2, 1:200 dilution, EMD Millipore, Burlington, MA, USA, RRID: AB_10807045), anti-Glutamine Synthetase (Müller glia, 1:200 dilution, Sigma-Aldrich, St. Louis, MO, USA; RRID: AB_259853), anti-Calbindin-D-28 K (cone photoreceptor cells, 1:500 dilution, Millipore Sigma, Burlington, MA, USA, RRID: AB_258818), anti-RPE65 (retinal pigment epithelium, 1:500 dilution, ThermoFisher, Waltham, MA, USA; RRID: AB_2181003), and anti-phospho Histone H3 (marker for mitosis, 1:500 dilution, EMD Millipore, RRID:AB_310177). Alexa fluor-conjugated secondary antibodies were used at a 1:1000 dilution (ThermoFisher, Waltham, MA, USA). For each antibody, n > 5 was used with 3–4 sections per sample.

Microscopy: A Nikon A1R confocal laser scanning microscope (Tokyo, Japan) (UNLV Confocal and Biological Imaging Core) was used to image Islet1, RPE65, and Glutamine Synthetase immunostained tissue sections. All other immunostained tissue sections were visualized via a Zeiss Axio Upright Imager M2 microscope (Danvers, MA, USA) with a Hamamatsu ORCA flash 4.0 monochromatic digital CMOS camera (Shizuoka, Japan. Images of whole animals were obtained using a ZEISS SteREO Discovery V20 microscope with an AxioCam MRc camera (Zeiss, Danvers, MA, USA). ZEN Image Analysis software (https://www.zeiss.com/microscopy/en/products/software/zeiss-zen.html (accessed on 27 February 2025)) and/or the open-source FIJI imaging software (v1.5) were used to analyze and/or process all acquired images [64].

Statistical Analysis: To compare eye regrowth, raw data from scoring were used. The comparison of two treatments was analyzed with the Mann–Whitney U test for ordinal data with tied ranks, using a normal approximation for large sample sizes. All other experiments were analyzed using a Student’s *t*-test.

## Data Availability

Data is contained within the article.
